# A Treatise on Poisons, in Relation to Medical Jurisprudence, Physiology, and the Practice of Physic

**Published:** 1845-05

**Authors:** 


*A Treatise on Poisons, in relation to Medical Jurisprudence, Physiology, and the Practice of Physic.* By ROBERT CHRISTISON, M.D., F. R. S. E., Professor of Materia Medica in the University of Edinburgh, Fellow of the Royal College of Physicians, etc. etc. etc. First American, from the fourth Edinburgh Edition. In two parts. 8vo. pp. 756. Barrington & Haswell. Philadelphia, 1845.

This first American Edition of Dr. Christison's work on Poisons, comes to us in two parts—the one in January, and the other in April—as the quarterly portions of the Select Medical Library, edited by Dr. Bell.

It would be a work of supererogation, at the present day, to speak of the merits of this work. It has long and justly been regarded as one of our standard authorities. The fourth Edinburgh Edition, of which the present is a reprint, was republished in that city in November last, (1844,) and on every subject the author has been careful to post up the latest information. We know, indeed, of no one who is more careful in this respect, evinces more judgment and discrimination in the selection of his materials, or is more scrupulously just in referring to those from whom he has derived them. In the republication of such a work, the Editor and Publishers have conferred a great favour upon the profession in this country.

